# Surface Glycans of Microvesicles Derived from Endothelial Cells, as Probed Using Plant Lectins

**DOI:** 10.3390/ijms25115725

**Published:** 2024-05-24

**Authors:** Ekaterina V. Slivka, Nadezhda V. Shilova, Ekaterina A. Obraztsova, Daria S. Kapustkina, Sergey V. Khaidukov, Alexey Yu. Nokel, Ivan M. Ryzhov, Stephen M. Henry, Nicolai V. Bovin, Eugenia M. Rapoport

**Affiliations:** 1 Shemyakin and Ovchinnikov Institute of Bioorganic Chemistry RAS, 16/10 Miklukho-Maklaya Str., Moscow 117997, Russia; slivkaekaterina6@gmail.com (E.V.S.); pumatnv@gmail.com (N.V.S.); imryzhov@gmail.com (I.M.R.); eugenia_rapoport@mail.ru (E.M.R.); 2 National Medical Research Center for Obstetrics, Gynecology and Perinatology of the Ministry of Health of the Russian Federation, 4 Oparina Str., Moscow 117997, Russia; 3 School of Engineering, Auckland University of Technology, Auckland 1010, New Zealand; shenry@kodebiotech.com

**Keywords:** extracellular vesicles, glycans, glycosphingolipids, endothelial cells, microvesicles

## Abstract

Glycans of MVs are proposed to be candidates for mediating targeting specificity or at least promoting it. In contrast to exosomes, glycomic studies of MVs are largely absent. We studied the glycoprofile of endothelial cell-derived MVs using 21 plant lectins, and the results show the dominance of oligolactosamines and their α2-6-sialylated forms as N-glycans and low levels of α2-3-sialylated glycans. The low levels of α2-3-sialosides could not be explained by the action of extracellular glycosidases. Additionally, the level of some Man-containing glycans was also decreased in MVs. Spatial masking as the causative relationship between these low level glycans (as glycosphingolipids) by integral proteins or proteoglycans (thus, their lack of interaction with lectins) seems unlikely. The results suggest that integral proteins do not pass randomly into MVs, but instead only some types, differing in terms of their specific glycosylation, are integrated into MVs.

## 1. Introduction

Extracellular vesicles (EVs) play an important role in cell–cell communication by transferring proteins, lipids, DNA, and mRNA from parent cells to target cells [1]. According to their size, EVs are classified as exosomes (30–100 nm), microvesicles (50–1000 nm), or apoptotic bodies (>1 μm); the latter not being involved in intercellular communication. Exosomes are EVs of endosomal origin; they are formed inside cells during the maturation of multivesicular bodies and are secreted upon fusion of multivesicular bodies with the plasma membrane. Microvesicles (MVs) are released via budding from the plasma membrane [1,2]. The following plasma membrane proteins are specific markers of MVs: arrestin domain-containing protein 1 (ARRDC1), tetraspanin CD73, and annexin I, while specific markers of exosomes are tetraspanins, including CD9, CD63, CD81, and the proteins TSG101 and Alix from the ESCRT group; ESCRT proteins promote the transfer of multivesicular bodies into the plasma membrane [3,4]. EVs are considered to be attractive tools for drug delivery, as the stability of their membrane facilitates safety for the molecules transported to the target cells. The interaction of EVs with receptors on target cells plays a significant role in the fate of the transferred molecules. Endogenous cellular lectins promote internalization via binding to glycans of EVs; for example, sialylated Le^x^ containing exosomes are engulfed by activated endothelial cells due to the binding of their E-selectin to glycan [5]. The uptake of exosomes by dendritic cells via binding of DC-SIGN lectin to Le^x^ was also shown in the same research. The DC-SIGN of dendritic cells facilitates the endocytosis of exosomes, produced by melanoma cells, by binding to mannose-containing glycans of exosomes [6], while siglec-9 mediates the engulfment of glioma-derived exosomes by dendritic cells via binding to α2-3- and α2-6-sialylated glycans [7]. These publications demonstrate the significance of the information about the glycan profile of EVs.

The application of mass spectrometry for the study of the glycoprofile of EVs is limited, since it does not provide information about the accessibility of glycans for recognition; nevertheless, the glycolipid composition can be determined using this approach [2,8]. In this regard, it has become more practical (although less structurally informative) to identify glycans in EVs using lectins, which is carried out using three methods: (1) flow cytometry [9,10,11]; (2) lectin arrays employing fluorescently labelled EVs [12,13,14,15]; and (3) the isolation of glycoprotein fractions from EVs and analysis by electrophoresis followed by lectin blotting [10,11]. To our knowledge, a comparative analysis of the results obtained using various methods has not been performed.

It should be emphasized that the most well-known investigations are devoted to the glycoprofiles of exosomes, while there is much less data on the glycoprofiles of MVs. Two research groups recently demonstrated that the glycoprofiles of MVs and their parent cells do not always match [10,11]. For example, α2-3-sialylated and fucosylated glycans present on WM115, WM266-4, and WM293 cells (melanoma origin) are only present on MVs derived from WM115 and WM266-4 cells [10]. Fucosylated glycans on T24 (bladder cancer) cells and HCV 29 (ureter cells) cells are absent in the MVs derived from them [11].

Information about the glycoprofiles of EVs derived from endothelial cells is only available for exosomes isolated from rat cells. The binding of four lectins, *Sambucus nigra* (SNA), *Maackia amurensis* (MAA), *Griffonia simplicifolia* I (GSL I), and *Helix pomatia* (HP), to exosomes from pulmonary microvascular endothelial cells (MVECs), pulmonary artery endothelial cells (PAECs), and aortic endothelial cells (AOECs), was analyzed using flow cytometry. It was shown that exosomes and parent AOECs are enriched by Galα-terminated and α2-3-sialylated glycans [9]. On the contrary, the level of Galα-terminated glycans was decreased in exosomes originating from MVECs as compared to parent cells; PAECs were enriched by α2-6 and α2-3-sialylated glycans, unlike exosomes derived from these cells. Our research focused on a lectin-defined study of the glycoprofile of MVs derived from endothelial EA.hy 926 cells. Based on this information, we can predict which blood cell lectins have the potential to participate in the uptake of specifically glycosylated MVs and examine whether this mechanism is utilized.

## 2. Results

### 2.1. Isolation and Imaging of MVs

**Figure 1:**
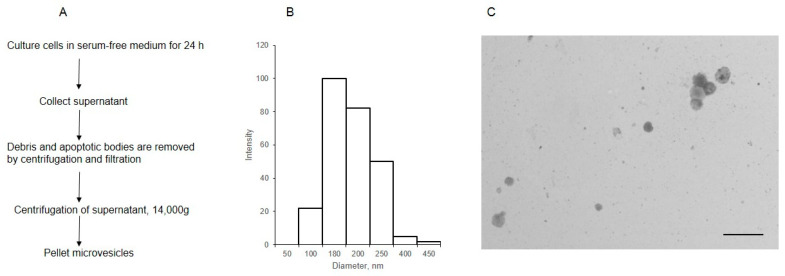
The scheme of isolation (**A**) and characterization of MVs derived from EA.hy 926 (**B**,**C**). The size distribution of MVs was determined by dynamic light scattering (**B**), the light intensity (arbitrary units) is plotted against the diameter, nm; (**C**) the morphology of MVs, as observed by transmission electron microscopy (inset bar = 1000 nm).

MVs were isolated from the cell supernatant by centrifugation, after the removal of debris and apoptotic bodies (Figure 1A). The analysis of MVs using dynamic light scattering showed that the size of most of the particles was within 180–250 nm (Figure 1B). The STEM image (Figure 1C) shows that the diameter of most MVs was 180 nm, aligning with the data previously obtained for MVs isolated from the same cells [16].

### 2.2. Test System for Glycan Profiling

In the first and second preparation versions, the binding of biotinylated lectin to MVs (MVs labeled as described in the Materials and Methods section, see Supplementary Figure S1) was not observed, as the MVs did not immobilize on the slides. In the third version, where the MVs were immobilized on poly-*L*-lysine, the signal from the MVs was confirmed, as described in Supplementary Figure S1; therefore, the immobilization on poly-*L*-lysine was further utilized.

Twenty-one lectins were used for glycoprofiling of the MVs, using a solid-phase assay. The typical glycans recognized by these lectins, according to data from the Consortium of Functional Glycomics (CFG) [17], are presented in Table 1; eight of them displayed reliable binding to MVs in the solid-phase assay.

### 2.3. Solid-Phase Assay MV Glycan Profile

**Figure 2:**
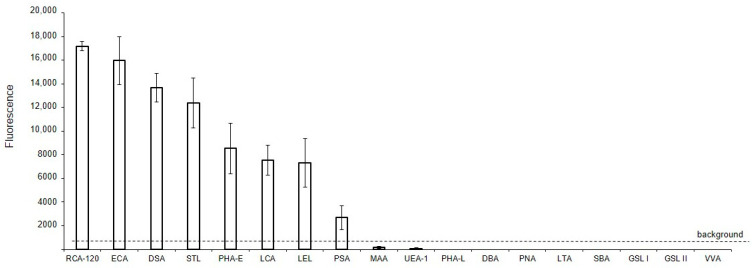
Solid-phase detection of lectin binding to MVs. MVs were derived from EA.hy 926 cells and immobilized on glass slides (solid-phase assay), incubated with biotinylated lectins, and then visualized with Str-Alexa Fluor 555, as described in the Materials and Methods section.

The binding of lectins to glycans of MVs derived from EA.hy 926 cells, immobilized on glass slides (solid-phase assay), is shown in Figure 2. It should be noted that the data shown in Figure 2 are qualitative or semi-quantitative for two reasons: first, it is incorrect to compare the binding of lectins with each other directly, since their affinities are unknown (and probably different) and, second, the different degree of biotinylation of these reagents adds uncertainty in regard to the interpretation of the results. Nevertheless, the presented data allows us to determine whether the binding of lectin to MVs is absent, as well as to compare the glycan profiles of MVs to the parent cells. Among the 21 lectins studied, the background signals were too high for six lectins, which recognize terminal Fucα (LTA and UEA I), GalNAcα (SBA, GSL I, VVA), and GlcNAcβ (GSL II) residues, to determine whether they reacted with MVs or not. Three other lectins, Jacalin, ConA, and SNA, bound directly to the glass slides, and so their results were excluded. The two lectins recognizing α2-3-sialylated (MAA), O- (PNA), and complex N-glycans (DBA), did not bind to MVs. Among the lectins expected to recognize Manα-containing glycans, only LCA and PSA bound to MVs, while PHA-L did not. The top eight lectins recognized oligolactosamine chains (RCA-120, ECA, DSA, and STL, see Supplementary Figure S3, Figure 2), substituted lactosamines (LEL), complex (PHA-E), and Man-rich N-glycans (LCA, PSA).

### 2.4. Glycan Profile of MVs According to Flow Cytometry

The binding of those lectins to MVs, which were unable to generate a result in the solid-phase assay, were analyzed using flow cytometry.

**Figure 3:**
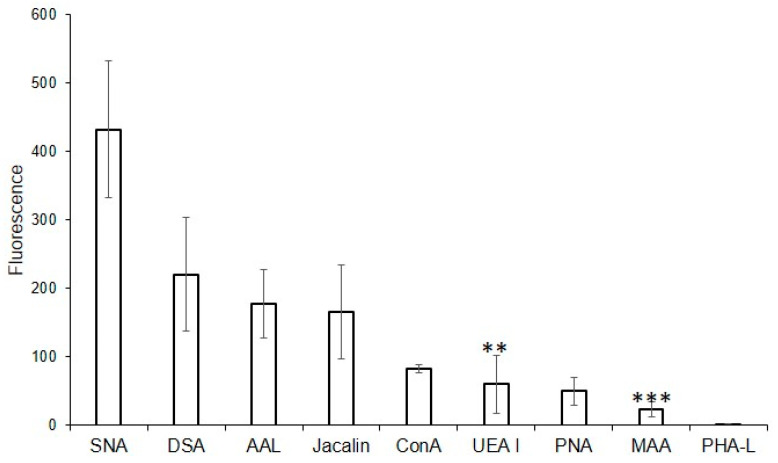
Flow cytometry detection of lectin binding to MVs. Results are from four experiments; error bars represent standard deviations. A mean fluorescence < 50 was considered as negative; *** *p* < 0.004, ** *p* < 0.004.

For this purpose, the following lectins were examined using flow cytometry: DSA (one of the eight best lectins in the solid-phase analysis) and non-binding MAA, UEA I, PHA-L, and PNA, while SNA, ConA, and Jacalin, due to the high background signals turned out to be unsuitable in the solid-phase analysis. Binding to MVs was not observed for PHA-L and MAA (Figure 3), confirming the results from the solid-phase assay. Among the lectins recognizing *O*-glycans, binding to MVs using flow cytometry was observed for Jacalin, but not for PNA (which also did not bind to MVs in the solid-phase assay). Lectins ConA and SNA, recognizing Manα-terminated and α2-6-Sia-containing glycans respectively, bound to MVs. UEA I displayed low binding. As UEA I is known to recognize only α1-2-fucosylated glycans, the binding of the AAL lectin (from *Aleuria aurantia*), which is known to display affinity with any fucosylated glycan (Table 1; [18]), was additionally studied. The binding of the AAL lectin to MVs (Figure 3), demonstrated that several fucosylated glycans are present in MVs. The combined data obtained from the flow cytometry and solid-phase assays were, in general, similar, namely oligolactosamines were revealed in the MVs, whereas α2-3-sialylated glycans were not detected (Figure 3).

**Figure 4:**
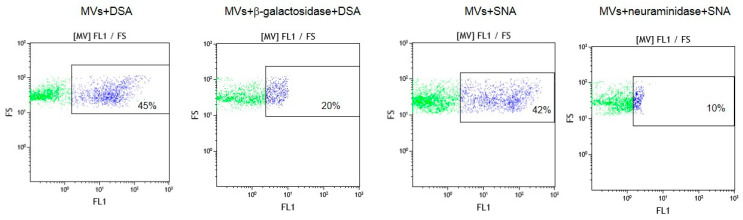
Flow cytometry detection of lectins DSA and SNA binding to glycosidase-treated MVs. MVs were incubated with either β-galactosidase or neuraminidase, washed and incubated with biotinylated lectins, as described in the Materials and Methods section. In the dot plots, forward scatter (FS) was plotted against the logarithm of fluorescence (FL1) and the number given in the rectangles (for the purple dots) indicate the percentage of lectin bound to MVs.

To confirm that oligolactosamines and α2-6-sialylated glycans were present in the MVs, they were treated with glycosidases, which cleave terminal Galβ or Neu5Acα (e.g., galactosidase and neuraminidase) residues. These enzyme treatments decreased the binding of the lectins recognizing oligolactosamines (DSA) and α2-6-sialylated glycans (SNA) to MVs (Figure 4).

### 2.5. Binding of Lectins to Parent EA.hy 926 Cells

**Figure 5:**
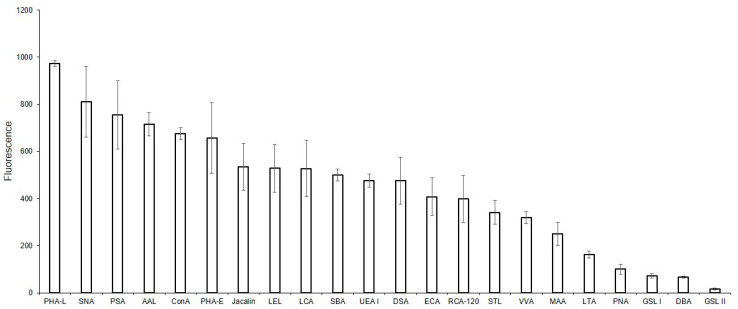
Flow cytometry binding of lectins to EA.hy 926 cells. Cells were incubated with biotinylated lectins followed by Str-FITC, as described in the Materials and Methods Section. Results shown include data from four experiments and error bars represent standard deviations. A mean fluorescence <50 was considered as negative.

To compare the glycoprofiles of MVs and the parent EA.hy 926 cells, the binding of the cells against the same for lectins was studied (Figure 5). We initially attempted to study the glycoprofile of the cells using the solid-phase assay, however consistent cell immobilization could not be attained. For this reason, only a flow cytometry assay was performed. The mean fluorescence reactions of the DBA, PNA, GSL-I, and GSL II lectins were below the threshold of 50 and were, therefore, considered as negative. Among the lectins recognizing Galα and GalNAcα-terminated glycans, binding was observed for SBA and VVA, but not for GSL I. Of the lectins showing affinity to *O*-glycans, Jacalin, but not PNA, bound to the cells. The MAA, SNA, AAL, and UEA I lectins all bound to the cells, confirming the presence of α2-3-, α2-6-sialylated, and fucosylated glycans on the cell surface. The ConA and PHA-L lectins, recognizing Man-containing glycans, and lectins with an affinity to oligolactosamines (RCA, ECA, DSA, STL, and LEL) bound to the MVs. Similarly, lectins recognizing Man-rich (PSA and LCA) or complex glycans (PHA-E) also bound to the cells.

### 2.6. Binding of Lectins to MVs Derived from Cells Treated with Glycosylation Inhibitors

**Figure 6:**
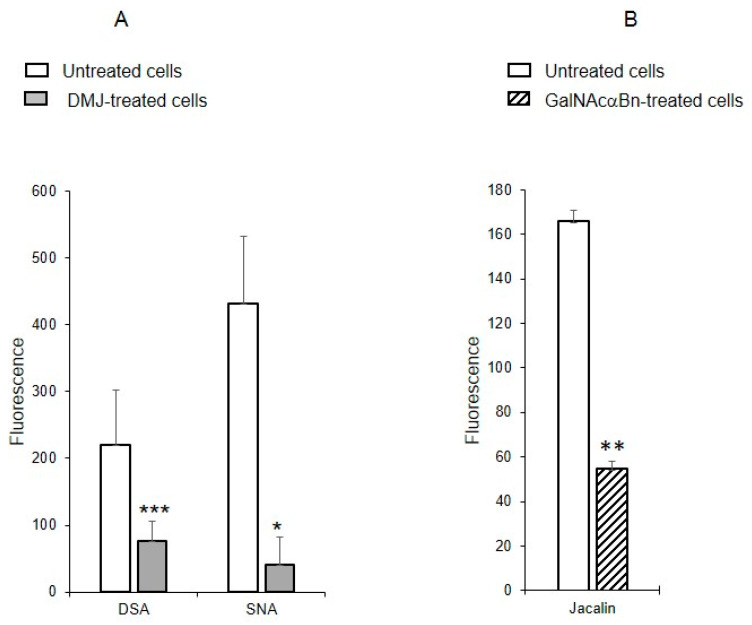
Binding of lectins to MVs derived from EA.hy 926 cells treated with inhibitors to *N*-glycosylation, DMJ (**A**) or *O*-glycosylation, GalNAcαBn (**B**). MVs were isolated from native cells and cells treated overnight with GalNAcαBn or DMJ. Flow cytometry results shown include data from two experiments and error bars represent standard deviations; *** *p* < 0.001, ** *p* < 0.01, * *p* < 0.1.

To determine whether the identified oligolactosamines and α2-6-sialylated glycans were present on glycoproteins (and if so, what type of glycoproteins), instead of just glycolipids, MVs were isolated from the cells treated with the reagent DMJ, which is known to block the biosynthesis of *N*-glycans [19], or with GalNAcαBn, which inhibits *O*-glycosylation [20]. The binding of DSA and SNA to MVs isolated from the cells with suppressed biosynthesis of *N*-glycans was dramatically decreased, compared to MVs from native cells (Figure 6A). The binding of Jacalin to MVs from cells with suppressed biosynthesis of *O*-glycans was also reduced as compared to native MVs, although not as pronounced as for *N*-glycans (Figure 6B). This data indicates that glycoproteins carrying both *N*- and *O*-glycans are present in MVs.

### 2.7. Binding of Lectins to MVs Derived from Cells after Depletion of Glycocalyx

To study whether the MVs have acquired proteoglycans and glycoproteins from the glycocalyx of the parent cells, MVs were obtained from cells with a depleted glycocalyx. The cells were treated simultaneously with collagenase and hyaluronidase, known to decompose collagen and proteoglycans, and then with trypsin to digest glycocalyx proteins. The degree of glycocalyx depletion was then visualized with lectin PHA-L. Glycocalyx was either not detected, or its thickness was significantly reduced (see Supplementary Figure S4).

**Figure 7:**
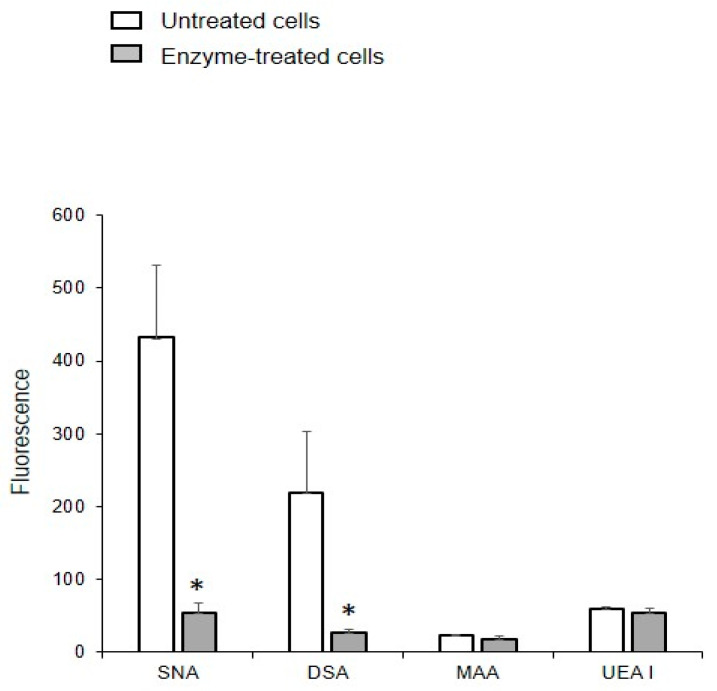
Binding of lectins to MVs released from the EA.hy 926 cells treated with enzymes. MVs were isolated from cells treated with collagenase, hyaluronidase, and trypsin. Flow cytometry results include data from three experiments; error bars represent standard deviations; * *p* < 0.1.

The binding of DSA and SNA to MVs obtained from glycocalyx-depleted cells was an order of magnitude lower than from MVs isolated from native cells (Figure 7), indicating that proteoglycans and glycoproteins are acquired from the glycocalyx of the parent cells. The enzymatic depletion of glycocalyx did not affect the binding of UEA I and MAA to MVs, which is probably due to the treatment not affecting the glycolipids.

### 2.8. Glycosidase Activity of EA. hy926 Cells and MVs

**Figure 8:**
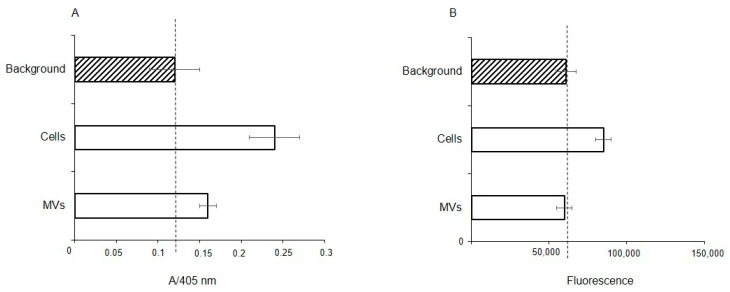
Fucosidase (**A**) and sialidase (**B**) activity in EA.hy 926 cells and MVs. Glycosidase activity was measured in membrane from cell and MV lysates using 4-nitrophenyl-α-L-fucopyranoside (NP-Fuc, (**A**), or 2-(4-methylumbelliferyl)-α-D-N-acetylneuraminic acid (MU-Neu5Acα, (**B**), respectively, as substrates. Absorbance of free 4-nitrophenol (**A**) or fluorescence of released free methylumbelliferone (**B**) are shown in arbitrary units ± standard deviations. Lysis buffer containing 1% Triton X-100 was used as a background for the absorbance (**A**) or fluorescence (**B**).

To determine whether extracellular glycosidases were responsible for the observed absence of Neu5Acα2-3 and the reduced levels of fucosylated glycans, the fucosidase and sialidase activities were measured in membrane fractions of the cells and MVs. The total protein concentration in both cases was the same at 28 μg/mL, thus a direct comparison is valid. Fucosidase activity was found in both the membrane fraction of the parent cells and MVs, but with enzyme activity about half for the MVs, (Figure 8A); in contrast, sialidase activity was seen only in the cell fraction (Figure 8B).

### 2.9. Binding of CTB to MVs

**Figure 9:**
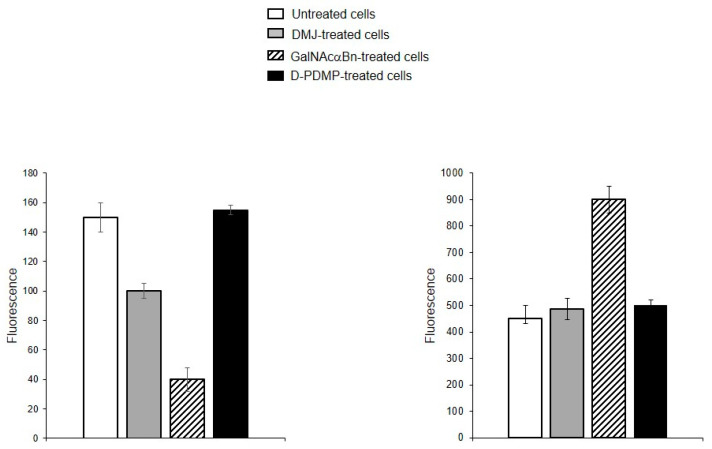
Flow cytometry detection of CTB binding to MVs derived from EA.hy 926 cells (**left panel**) and parent cells (**right panel**) treated with DMJ, GalNAcαBn, or D-PDMP.

In addition to plant lectins, the binding of cholera toxin B (CTB), which is known to bind ganglioside GM1 [21,22,23], was tested. The accessibility of the sialic acid residue in GM1 differs from other α2-3-sialylated glycans due to the GalNAc residue located in close proximity (at the Gal-4-OH), and as a consequence GM1 does not interact with all Siaα2-3-binding lectins, this is also true for SNA (data from the Consortium for Functional Glycomics). CTB, as seen in Figure 9, interacts with MVs, but since CTB has two carbohydrate-recognizing sites, one for GM1 and the second for glycoproteins [23], we could not confidently assign the observed interaction to GM1. Therefore, MVs obtained from cells treated with DMJ, GalNAcαBn, or D-PDMP, the latter blocking glycosphingolipid biosynthesis [24], were tested. This time the binding of CTB to MVs obtained from cells with blocked glycosylation of glycoproteins was reduced, especially for *O*-glycosylation (Figure 9, left panel). At the same time, the blocking of GSL biosynthesis in cells did not affect the binding of CTB to MVs (Figure 9, right panel). This was interpreted as CTB reacting with MVs bound to glycoproteins and not to the glycolipid GM1. A different pattern of CTB binding was found with the parent cells, namely none of the three types of biosynthesis blocking abolished binding (see Supplementary Figure S5), which was interpreted as CTB binding to both glycoproteins and GM1.

## 3. Discussion

**Figure 10:**
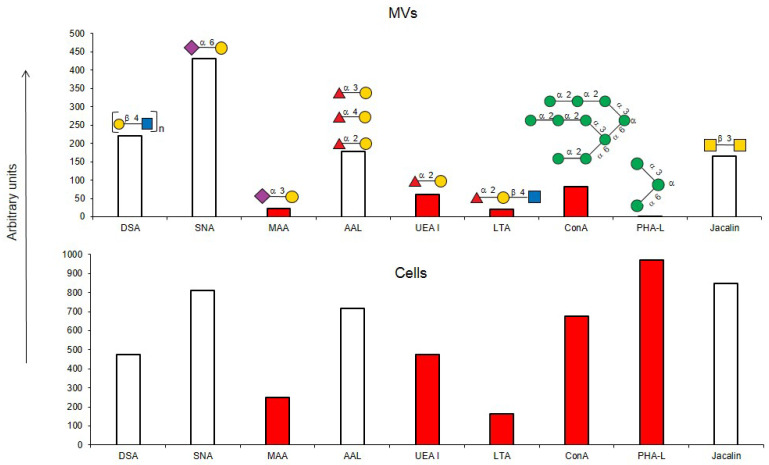
Comparison of lectin glycoprofiles of parent EA.hy 926 cells and MVs obtained from them, according to the lectin assays (cumulative data from Figure 2, Figure 3 and Figure 5). The structure of the primary ligand detected by the lectin is indicated in SNFG format (https://www.ncbi.nlm.nih.gov/glycans/snfg.html accessed on 26 February 2024) found at (http://csdb.glycoscience.ru/database/core/wizard.html accessed on 26 February 2024) using the Carbohydrate Structure Databases, found at http://csdb.glycoscience.ru/database/core/wizard.html accessed on 16 March 2024. The results which show significant variance between the parent cell and the MV are colored red.

If one of the functions of EVs is to transfer information, then their absorption by receiving cells needs to be selective rather than stochastic, i.e., there must be a mechanism for targeting EVs to specific cell types. Endogenous mammalian lectins on target cells are potential mediators of selective recognition; however, knowledge on the involvement of cellular lectins in this process is limited, particularly due to insufficient information about the glycan profile of EVs. Most known research provides data for exosomes, while the glycoprofile of MVs is less well investigated. In this work, we used known research lectins to glycoprofile MVs and compared them to their parent cell, assuming that specific glycosylation differences in the MVs membrane could potentially relate to targeting functions. A comparative analysis of the glycan profiles of the parent cells and the MVs derived from them is presented in Figure 10. Due to the dissimilarity of the membranous entities, a strict quantitative comparison of the reactions of lectins with MVs compared with their parent cells is not possible; however, a comparison between the glycoprofiles of the same entity (Figure 10) allowed for several conclusions. First of all, the glycosylation (or more precisely the lectin-binding profiles) of MVs and parental cells are similar, but not identical. The most obvious and dramatic difference between MVs and their parent cells is with respect to the PHA-L lectin. There is no doubt that PHA-L binds to the mannose-containing glycoprotein *N*-chains, but since the lectin ConA also detects the trimannose motif Manα1-6(Manα1-3)Man on the MVs, it is not possible to attribute the binding of PHA-L to a specific *N*-chain on the MV. This observation is further complicated by the poorly defined specificity of PHA-L [19] and requires further evaluation with an extended range of mannose-rich glycoprotein *N*-chains probes in order to define the glycan(s) involved. The next most significant difference is the lower levels of α2-3-sialylated glycans in MVs, as seen with MAA. If we assume that the content of lactosamine chains (as measured by DSA binding) in MVs and cells is similar and use it as a reference standard, then it appears that MAA binds to MVs about five times less than with parent cells.

Typically, α2-3 sialylation of the cells is represented primarily by complex type glycoprotein chains, and our results on glycosylation inhibition (see above) are in agreement with this, although the glycosphingolipid α2-3-sialylated ganglioside GM1 is expected to be present, as it is an obligatory component of the lipid raft. Sialylation was detected by the MAA lectin, but as this lectin is unable to detect some variants of α2-3-sialylated glycans, in particular the GM1 ganglioside [8,25], we therefore further examined the binding of MVs and parent cells to cholera toxin B (CTB), which recognizes GM1 [23], and found its interaction with both entities. However, as CTB is also known to bind to asialo glycans of glycoproteins [22,26], it does not allow us to conclusively claim that GM1 (and, accordingly, lipid rafts) are present on MVs. One possible explanation for the lower levels of α2-3 sialic acid glycans observed in MVs, is that α2-3 sialosides are present at a higher level than observed, but are sterically hindered from recognition, for example by cholesterol [27,28]. Specific *cis* masking by siglecs can be excluded, since siglecs are not expressed on parent endothelial cells [29]. A less likely explanation is that there is nonspecific shielding of α2-3-sialylated glycans in glycosphingolipids by integral proteins and proteoglycans, but this is questionable as it does not explain the lower levels of α2-3-sialosides in the glycoproteins of MVs; the loosening of the glycocalyx did not lead to unmasking and it is difficult to reconcile that only α2-3-sialylated glycans are physically shielded, while α2-6-glycans are not.

An alternative explanation for the low levels of α2-3-sialylated glycans in MVs, is that there is a mechanism for selecting a limited cohort of certain glycoconjugates (lipids and/or proteins) during the formation of MVs [12,13,25,30]. Such a mechanism for glycosphingolipids is supported by their known tendency to homo cluster in the plasma membrane [31,32], although a specific mechanism for glycoprotein selection is unknown. It is of note that the size and thickness of the glycocalyx of MVs (or more correctly the (glyco)protein corona of the type known for liposomes) is much less than the typical glycocalyx of endothelial cells. Additionally, from our results it can be seen that the glycosylation profile (i.e., glycocalyx) of the MVs is different in regard to the glycan composition when compared to the glycocalyx of the parent cells, in particular concerning its α2-3-sialylated glycans. CD44, VCAM-1, ICAM-1, integrins, and CD31 are expressed on EA.hy 926 cells [33]. With respect to the α2-6-sialylated glycans, it can be reasonably assumed that those glycoproteins transferred to MVs have only α2-6 sialylation (as α2-3-sialylated glycans were essentially absent). We tested for CD31, which is heavily glycosylated with α2-6-sialylated complex-type glycans [34] and it was detected in the MVs with the corresponding antibodies. We also excluded the action of endo-α2-3 sialidase, as we did not detect sialidase activity in MVs, and only minor activity was detected in the membrane fraction of parent cells.

With respect to fucosylated glycan binding AAL and UEA-I lectins, like α2-3-sialylated glycans, they showed a decrease in the level of α1-2 fucosylated glycans in the MVs (Figure 10). However, this difference was not considered reliable within the limitations of the quantitative comparison between the flow cytometry of cells and MVs. Additionally, the action of membrane-bound fucosidases [35] present in both the cells and MVs could not be excluded, although the effect if any was probably not significant as the enzyme was present in both cells and MVs.

In summary, MVs and the parent cells they are derived from differ in regard to their lectin-defined glycoprofiles. The most significant conclusive feature is the decrease in MVs of their content of α2-3-sialylated glycans relative to their α2-6-sialylated and polylactosamines glycans. Similar results have been observed for exosomes [12,13], despite different mechanisms of formation related to these extracellular vesicles. The extent that the different glycosylation patterns identified here for MVs formed from EA.hy 926 cells will be reproduced in other cell types is unknown, and comparisons of the data in the literature are limited because most publications are devoted to exosomes [8,12,13,15,25,30].

Although our results show that MVs have a more limited glycan profile than the parent cell, this may in itself create a novel targeting pattern, as galectin binding to oligolactosamines and siglec binding to α2-6-sialylated glycans should be enhanced on MVs [36].

## 4. Materials and Methods

### 4.1. Materials

DMEM-F12, glutamine, and DAPI (4′,6-diamidino-2-phenylindole) were from Invitrogen Co. (Carlsbad, CA, USA). Fetal calf serum (FCS), carbohydrate-free bovine serum albumin (BSA), streptavidin-FITC conjugate (Str-FITC), Tween-20, 4-nitrophenyl-α-L-fucopyranoside (4NP-Fuc), 2-(4-Methylumbelliferyl)-α-D-N-acetylneuraminic acid sodium salt hydrate (MU-Ne5Ac), benzyl-2-acetoamido-2-deoxy-α-D-galactopyranoside (GalNAcαBn), 1-Deoxymannojirimycin hydrochloride (DMJ), d-*threo*-1-phenyl-2-decanoylamino-3-morphlino-1-propanol (D-PDMP), neuraminidase from *Vibrio cholera*, β-galactosidase from *Aspergillus oryzae*, ethanolamine, and anti-rabbit and anti-mouse IgG conjugated with FITC were from Sigma (St-Louis, MN, USA). Streptavidin conjugated with Alexa Fluor 594, or Alexa Fluor 555 (Str-Alexa Fluor 594, or Str-Alexa Fluor 555), conjugate of cholera toxin B with Alexa Fluor 488 (CTB-Alexa Fluor 488), were from Thermo Fischer Scientific (Eugene, OR, USA). The biotinylated lectins listed in Table 1 were obtained from Vector Laboratories (Burlingame, CA, USA). NHS-activated glass slides H were from Schott Nexterion (Jena, Germany).

Synthetic glycolipid FSL (Function-Spacer-Lipid) constructs (see Supplementary Figure S2) were all based on the lipid DOPE (1,2-O-dioleoyl-*sn*-glycero-3-phosphatidylethanolamine). FSL-H (type 2) and FSL-Fluo constructs, synthesized as described in [37], were from GlycoNZ, Auckland, NZ (0089-FSL). All other reagents were from Reachem Corp. (Moscow, Russia).

### 4.2. Cell Culture

The EA.hy 926 cells were a gift from Dr. C.-J. Edgell (Chapel Hill, NC, USA). This is an immortalized cell line derived from HUVECs and human lung carcinoma cell line A549 [33], which retains the main functional characteristics of endothelial cells as migration and tube formation. The EA.hy 926 cells were cultured in DMEM-F12 supplemented with 10% FCS and 2 mM glutamine at 37 °C in a humidified atmosphere of 5% CO_2_.

### 4.3. Isolation of EA.hy 926 Cell-Derived MVs

To avoid contamination with vesicles originating from the calf fetal serum, the cells were cultivated in a serum-free medium. The supernatant from the EA.hy 926 cells cultured in a serum-free DMEM-F12 medium for 24 h was collected and centrifuged at 1000× *g* for 30 min at 4 °C to remove debris. The obtained supernatant was filtered through a 0.45 μm filter (Membrane Solution, Shanghai, China), and centrifuged at 14,000× *g* in a JA-20 rotor (Beckman Coulter Life Sciences, Indianapolis, IN, USA) for 40 min at 4 °C to create MVs in pellet form. The fraction containing MVs was washed twice in 200 μL PBS, under the same conditions. The obtained MVs were frozen at −70 °C until use. The MVs with inserted FSL constructs were used immediately.

### 4.4. Scanning Transmission Electron Microscopy

The size of the MVs was determined by scanning transmission electron microscopy (STEM). Briefly, the MVs were adsorbed on a 400 mesh formvar/carbon copper grids from a 5 μL drop containing 1.2 × 10^8^ of MVs, for 3–5 min. The samples were then negatively stained by double repeated 1 min immersions in 1% uranyl acetate. Imaging was performed by a Zeiss Merlin scanning electron microscope (Carl Zeiss, Oberkochen, Germany), operated in transmission mode. The images obtained were processed by ImageG software (https://imagej.net/ij, v1.4.3.x accessed on 1 January 2006) to analyze the MV size.

### 4.5. Glass Slides Preparation

Three different glass slide preparation procedures were used for optimization. In the first version, the MVs were printed using a SciFlexArrayer S5 (Scienion, Berlin, Germany) non-contact robotic printer, from 300 mM sodium phosphate, containing 0.001% Tween-20, on NHS-activated glass slides *H* (Schott Nexterion, Jena, Germany) as 14 replicates; the relative humidity was 50% and the drop volume was ~0.9 pL. The content of the MVs was ~1.7 × 10^3^ particles per spot. In the second version, the MVs were printed from 10 mM phosphate buffer saline containing 0.005% Tween-20 on NHS-activated glass slides *H* using a contact manual glass slide arrayer replicator (V&P Scientific, San Diego, CA, USA) as 24 replicates; the relative humidity was 20% and the drop volume was ~6 nL The content of the MVs was 3.6 × 10^5^ per spot. In the third version, the MVs were dropped onto glass slides covered with poly-*L*-lysine (Polysciences Inc., Warrington, RA, USA) as 4 replicates, with a drop volume of ~0.1 μL. The content of the MVs was 3.6 × 10^5^ per spot. In all three versions, the slides with MV spots were incubated for 1 h at room temperature and a relative humidity of 75% and kept in a desiccator.

### 4.6. Study of Interaction between MVs and Lectins on Glass Slide

The slides with MVs (first and second versions, see above) were kept in a blocking buffer containing 25 mM ethanolamine, 100 mM boric acid, 0.2% (*v*/*v*) Tween-20, at pH 8.5, under continuous stirring at room temperature for 90 min and then washed 2 times with PBS, containing 0.05% (*v*/*v*) Tween-20 (PBS-0.05%). Poly-*L*-lysine (third version) covered glass slides with the MVs were kept in a blocking buffer containing 1% BSA in PBS under continuous stirring for 60 min at room temperature. After blocking, the slides were washed twice with PBS 0.05% and incubated with lectins (20 μg/mL) in PBS, containing 1% BSA and 0.1% Tween-20 at 37 °C under continuous stirring for 1 h. The slides were then washed five times with PBS 0.05% and incubated with Str-Alexa Fluor 555 (dilution 1:1000 in PBS, containing 1% BSA and 0.1% Tween-20) under the same conditions. At the final stage, the slides were washed twice in PBS 0.05%, then washed with double-distilled water, dried by centrifugation and then scanned with an InnoScan 1100 AL fluorescence reader (Innopsys, Carbonne, France), at resolution 10 µm. The images were processed using ScanArray Express 4.0 and Microsoft Excel 2013 (15.0.4569.1504) MSO (15.0.4569.1506 software. The results were presented as the median of relative fluorescence units (RFU), with the median absolute deviation (MAD). A signal with a fluorescence intensity exceeding the signal from the ligand-free surface by a factor of five was considered to be significant.

### 4.7. Insertion of FSL into MVs

The MVs were washed twice with DMEM-F12 without additives and dried by centrifugation at 14,000× *g* for 40 min at 4 °C, then incubated with FSL constructs (final concentration 5 μM) for 1 h at 37 °C under stirring on a Rotamix RM-1 (Elmi, Latvia), and washed twice with PBS and dried by centrifugation under the same conditions.

### 4.8. Binding of Plant Lectins to Cells and MVs, Flow Cytometry

The MVs were washed twice with PBS, incubated with biotinylated lectin (20 μg/mL) at 4 °C for 30 min, and then with Str-FITC (1:50 dilution in PBS) under the same conditions. Finally, the MVs were washed twice with PBS and dried by centrifugation at 14,000× *g* at 4 °C for 40 min.

The adherent cells were detached with Versene solution (PBS containing 0.02% EDTA), washed three times with PBA (PBS containing 0.02% BSA) and dried by centrifugation at 90× *g*, 4 °C for 3 min, and incubated with biotinylated lectins, as described above. The cells were washed three times with PBA. After washing, the MVs or cells were transferred into a tube containing 2 mL of PBS. Flow cytometry was performed at room temperature using a FACScan instrument (Becton-Dickinson Co, Franklin Lakes, NJ, USA), equipped with the FlowJo software (V10.5.3), or an FC500 cytofluorimeter (Beckman Coulter, Miami, FL, USA) equipped with the Kaluza 1.3 software. The MVs were gated against a PBS background. The fluorescence shown in the figures was calculated as [(F_i_/F_0_) × 100] − 100, where F_i_ is the geometric mean of the fluorescence intensity of the cells or MVs after incubation with lectin and Str-FITC, and F_0_ is the geometric mean of the fluorescence intensity of the cells or MVs stained only with Str-FITC.

To confirm that α2-6-sialylated glycans and oligolactosamines were present in MVs, the binding of SNA or DSA was carried out on the MVs pretreated with neuraminidase (1 U/mL) or β-galactosidase (0.6 U/mL) at 37 °C for 24 h.

### 4.9. Measurement of Fucosidase or Sialidase Activity in EA.hy 926 Cell-Derived MVs

The membrane fractions of cells and MVs were obtained as described above. Briefly, the cells (2 × 10^6^) or MVs (1 × 10^7^) were re-suspended in 100 μL of potassium phosphate solution (50 mM KH_2_PO_4_, 50 mM K_2_HPO_4_, pH 6) containing 1% (*v*/*v*) Triton X-100 (100 μL) and kept on ice for 40 min, then centrifuged as before, and the supernatant collected. To the obtained fractions (50 μL) were added 50 μL of 0.2 M acetate buffer (pH 4.5) containing 4NP-Fuc (50 μM), or MU-Neu5Acα (0.2 mM); the reaction mixture was incubated in a 96-well plate for 90 min at 37 °C. The presence of free 4-methylumbelliferone (sialidase activity) in the samples of the fractions (100 μL) was measured using a spectrofluorometer (Wallac 1420 Multilabel Counter, Perkin Elmer, Waltham, MA, USA), excitation at 365 nm, emission at 450 nm. The absorbance of 4-nitrophenol (fucosidase activity) was measured using the same spectrometer as the mean value of the optical density at 405 nm. The protein absorbance at 280 nm in the cells or MVs lysate was measured using a Nanodrop 2000c (Thermo Fisher Scientific, Waltham, MA, USA), and the protein concentration was calculated using Nanodrop 2000 1.6 software, with each measurement conducted in triplicate.

### 4.10. Isolation of MVs Derived from EA.hy 926 Cells, Treated with Inhibitors of Glycosylation

The EA.hy 926 cells were grown to confluence in the medium DMEM-F12-10% FCS. DMJ (final concentration 10 μg/mL) or GalNAcαBn (final concentration 2 mM), or D-PDMP (final concentration 10 μM) were added to the cell monolayer and left for 24 h, or 72 h at 37 °C in a 5% CO_2_ atmosphere. After the growth medium was removed, the cells were left in the serum-free DMEM-F12 medium for 24 h before MV isolation (as described above).

### 4.11. Isolation of MVs Derived from EA.hy 926 Cells with Depleted Glycocalyx

The cells were grown in flasks (75 cm^2^). After washing with DMEM-F12 without additives, hyaluronidase (2 U/mL) and collagenase (0.15 mg/mL) were added to the cell monolayer and incubated overnight at 37 °C in a humidified atmosphere of 5% CO_2_. The next day the cells were collected, washed three times with PBS, and incubated with a Trypsin solution (0.25% in PBS) for 15 min at 37 °C in 5% CO_2_. The cells were then washed with DMEM-F12 without additives and left in the same medium for 24 h before MV isolation.

To visualize the glycocalyx, the cells were collected, washed with PBA, and incubated with biotinylated PHA-L (10 μg/mL in PBA), followed by Str-Alexa-Fluor 594 (dilution 1:200 in PBA) at 4 °C for 20 min. A microscope mounting mixture containing 2.4 g of Mowiol 4-88, 6 g of glycerol, 6 mL of water and 12 mL of 0.2 M Tris-HCl (pH 8.5) was placed on a microscope slide, followed by cell suspension (10 μL). Images were obtained with a confocal microscope, Nikon Eclipse TE-2000-E (Nikon, Minato city, Japan), and analyzed with ImageJ v1.4.3.x accessed on 1 January 2006 software. At least ten randomly selected cells were analyzed.

### 4.12. Binding of Cholera Toxin B to MVs

The MVs were obtained from native cells, or glycosylation-blocked cells. The MVs were washed with PBS and incubated with CTB-Alexa Fluor 488 (10 μg/mL) on ice for 20 min. Finally, the MVs were washed twice with PBS, and analyzed using flow cytometry, as described above.

### 4.13. Statistical Analysis

Data represent means +/− standard deviation. The unpaired Student’s *t*-test for statistical analysis of the results was used.

## Figures and Tables

**Table 1:** Lectins used in this study, and the glycans to which they bind, according to CFG data. Lectins displayed binding to MVs are marked in grey. The three lectins marked with * displayed binding non-specifically to the glass slides, and those lectins along with the lectin marked # were only analyzed using flow cytometry. The structure of glycans was presented in the symbol nomenclature for glycans (SNFGs) format (https://www.ncbi.nlm.nih.gov/glycans/snfg.html accessed on 26 February 2024) using Carbohydrate Structure Databases, 16 March 2024 found at http://csdb.glycoscience.ru/database/core/wizard.html.

№	Source	Short Name	Typical Glycans Bound by This Lectin (CFG Data)
1	*Ricinus communis* seeds	RCA-120	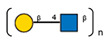
2	*Erythrina cristagalli* seeds	ECA	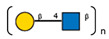
3	*Datura stramonium* seeds	DSA	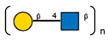
4	*Solanum tubersolum* potato	STL	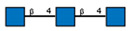
5	*Phaseolus vulgaris* seeds	PHA-E	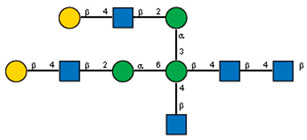
6	*Lens culinaris* seeds	LCA	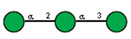
7	*Lycopersicon esculentum* tomato	LEL	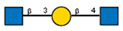
8	*Pisum sativum* peas	PSA	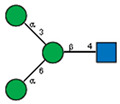
9	*Phaseolus vulgaris* seeds	PHA-L	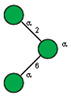
10	*Arachis hypogaea* peanut	PNA	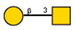
11	*Maackia amurensis* seeds	MAA	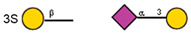
12	*Ulex europaeus* seeds	UEA I	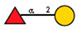
13	*Lotus tetragonolobus* seeds	LTA	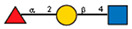
14	*Vicia villosa* seeds	VVA	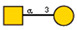
15	*Griffonia simplicifolia* seeds	GSL I	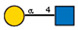
16	Lectin II from seeds of *Griffonia simplicifolia*	GSL II	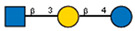
17	*Glycine max* soybean	SBA	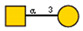
18	*Dolichos biflorus* seeds	DBA	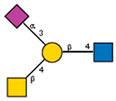
19	*Sambucus nigra* elderberry bark	SNA *	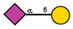
20	*Artocarpus integrifolia* seeds	Jacalin *	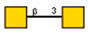
21	*Canavalia ensiformis* jack-bean	Con-A *	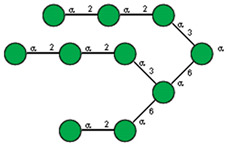 and other mannose-rich N-glycans
22	*Aleuria aurantia* orange peel fungus	AAL #	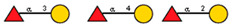
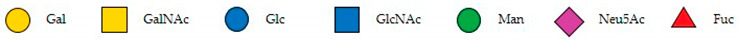			

## Data Availability

Data are contained within the article and Supplementary Materials.

## Supplementary Materials

The following supporting information can be downloaded at: https://www.mdpi.com/article/10.3390/ijms25115725/s1.
