# Supplementary material for: Surface Glycans of Microvesicles Derived from Endothelial Cells, as Probed Using Plant Lectins
Source: Int J Mol Sci. 2024 May 24;25(11):5725. doi: 10.3390/ijms25115725 (PMC11171894; doi:10.3390/ijms25115725)
Supplement: Supplementary file 1 [file ijms-25-05725-s001.zip › ijms-3000572-supplementary.pdf]

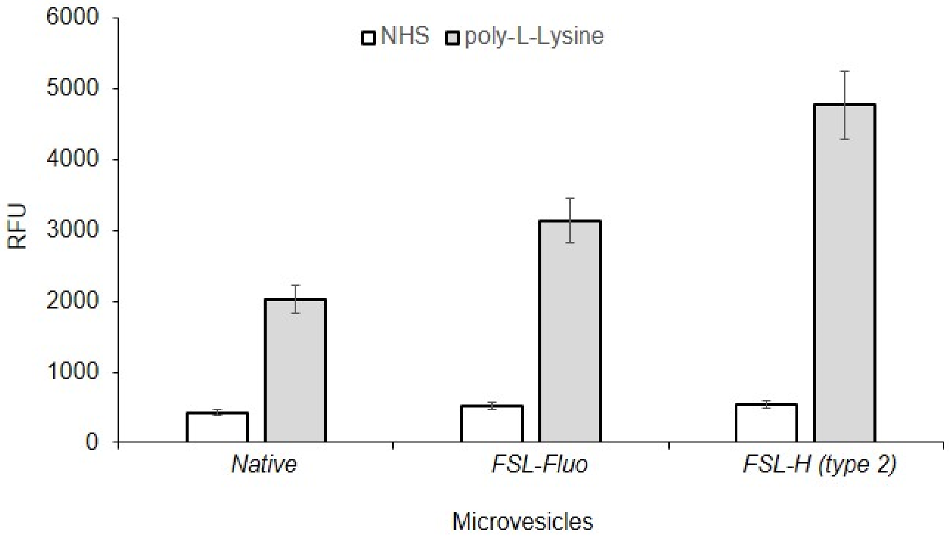

Figure S1. Comparative analysis of MV immobilization on NHS-activated or poly-*L*-lysine coated glass slides. The following MVs were immobilized: native, modified with FSL-Fluo, and modified with FSL-H(type 2) trisaccharide. H(type 2) in composition of MV was detected with lectin UEA I (*Ulex europaeus I*), which recognizes Fucα1-2Galβ (Table 1). The significant fluorescence signals from MVs were observed only in the case of the immobilization on glass slide coated with poly-*L*-lysine. Structures of FSLs are shown in Supplementary Figure S2. MVs native, or with inserted FSL (X axis) were plotted against RFU (Y axis).

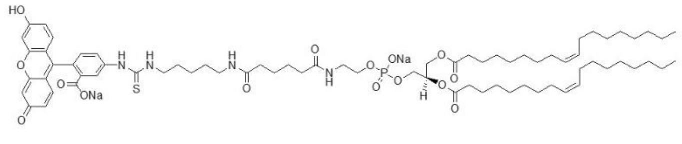

FSL-Fluo

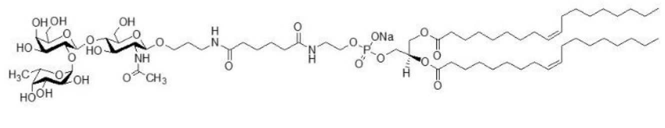

FSL-H (type 2)

Figure S2. Structures of FSL-Fluo and FSL-H (type 2)

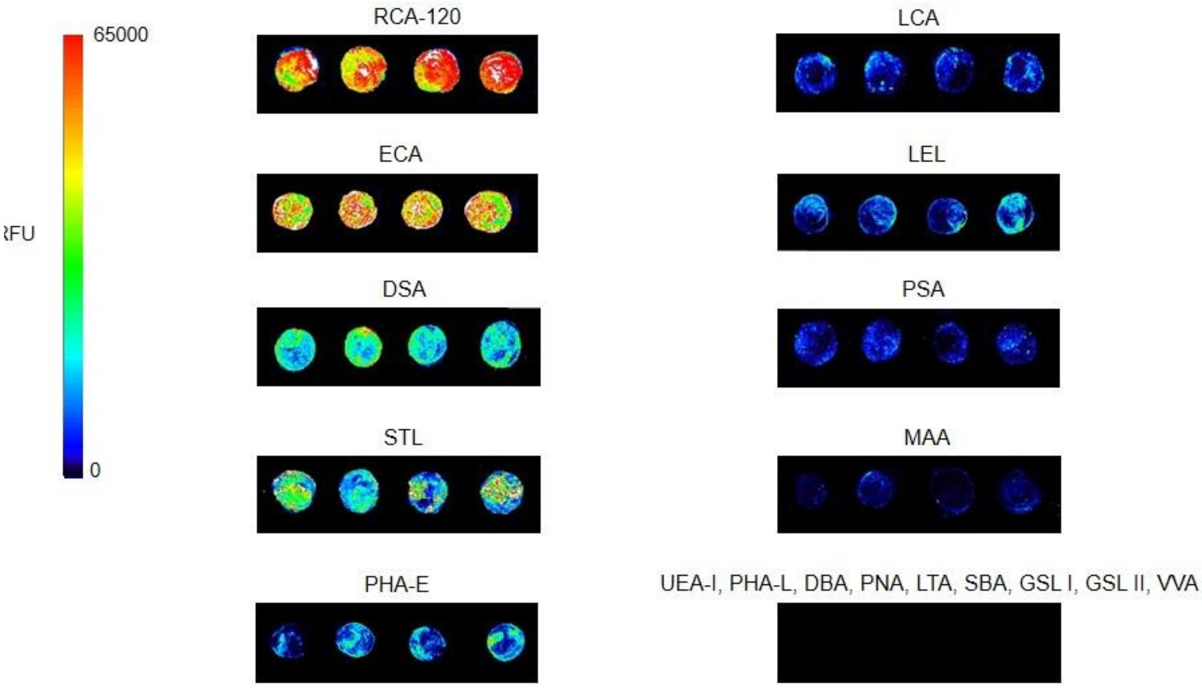

Figure S3. The binding of plant lectins to microvesicles immobilized on a glass slide. Spots are shown in a rainbow color scale, RFU, relative fluorescence units

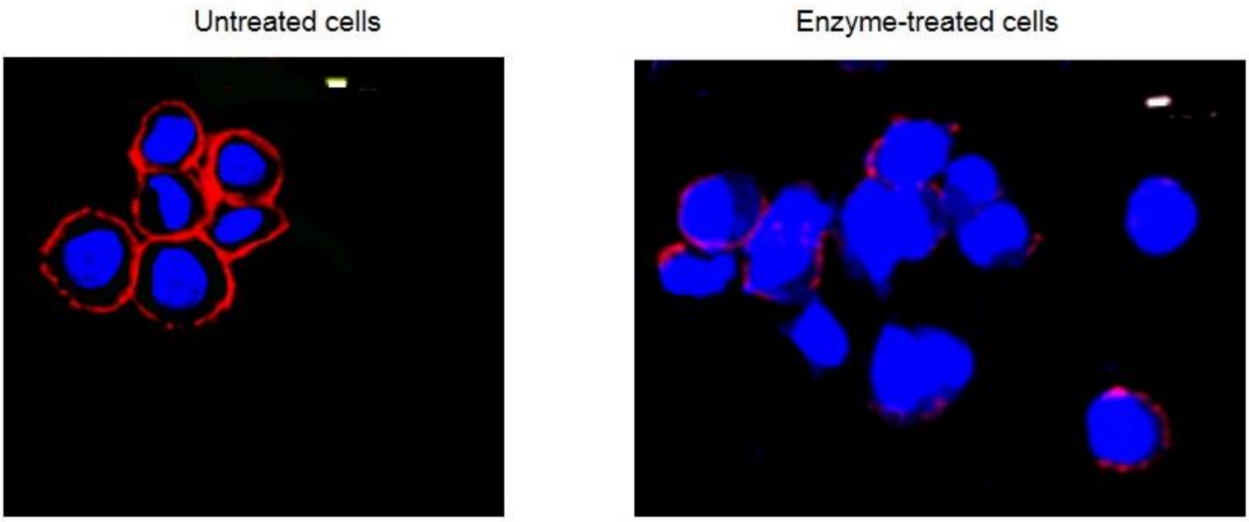

Figure S4. Confocal microscopy of PHA-L binding to EA.hy 926 cells after enzymatic depletion of the glycocalyx. Cells were enzyme treated with collagenase, hyaluronidase and trypsin, as described in Materials and Methods. The glycocalyx is stained red with biotinylated PHA-L-streptavidin-Alexa Fluor 594. Nuclei are stained blue with DAPI The white bar inset top right corresponds to 5 μm.

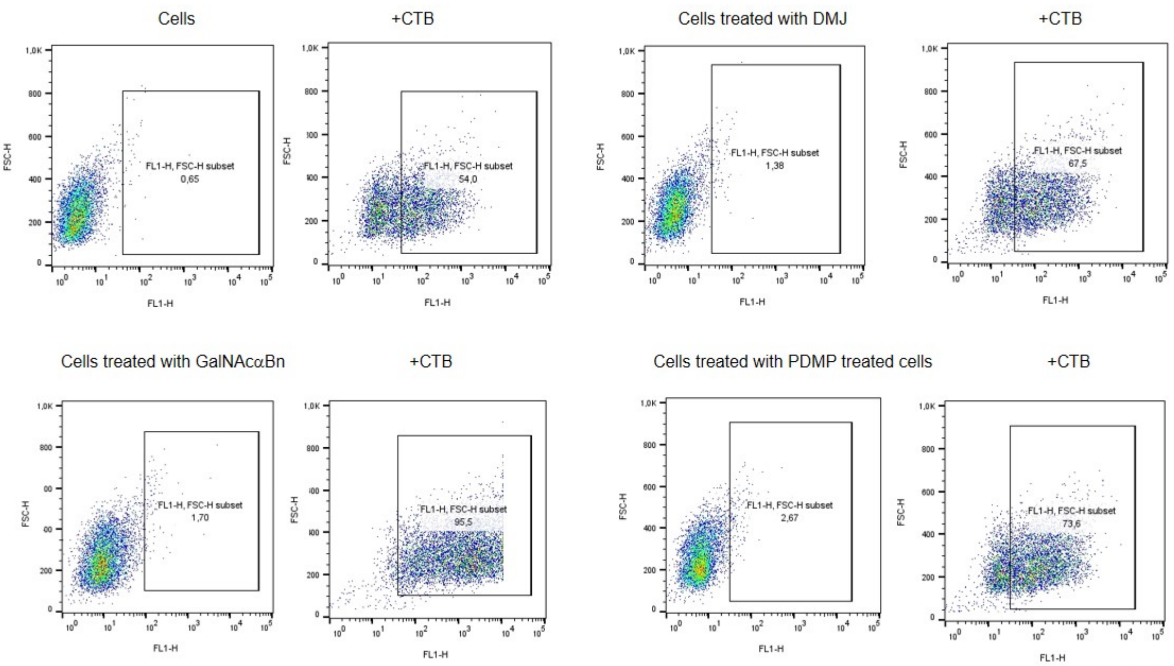

Figure S5. Binding of CTB to MV derived from EA.hy 926 cells treated with DMJ, or GalNAcαBn, or D-PDMP, flow cytometry data. MV were isolated from native cells and cells treated for 72h with GalNAcαBn, or DMJ, or D-PDMP. In the dot-plots presented, forward scatter (FS) was plotted against the logarithm of fluorescence (FL1), the number given in rectangle represents the percentage of CTB-bound Mv.
